# In-Cell NMR Characterization of the Secondary Structure Populations of a Disordered Conformation of α-Synuclein within *E. coli* Cells

**DOI:** 10.1371/journal.pone.0072286

**Published:** 2013-08-26

**Authors:** Christopher A. Waudby, Carlo Camilloni, Anthony W. P. Fitzpatrick, Lisa D. Cabrita, Christopher M. Dobson, Michele Vendruscolo, John Christodoulou

**Affiliations:** 1 Institute of Structural and Molecular Biology, University College London and Birkbeck College, London, United Kingdom; 2 Department of Chemistry, University of Cambridge, Cambridge, United Kingdom; Università di Napoli Federico II, Italy

## Abstract

α-Synuclein is a small protein strongly implicated in the pathogenesis of Parkinson’s disease and related neurodegenerative disorders. We report here the use of in-cell NMR spectroscopy to observe directly the structure and dynamics of this protein within *E. coli* cells. To improve the accuracy in the measurement of backbone chemical shifts within crowded in-cell NMR spectra, we have developed a deconvolution method to reduce inhomogeneous line broadening within cellular samples. The resulting chemical shift values were then used to evaluate the distribution of secondary structure populations which, in the absence of stable tertiary contacts, are a most effective way to describe the conformational fluctuations of disordered proteins. The results indicate that, at least within the bacterial cytosol, α-synuclein populates a highly dynamic state that, despite the highly crowded environment, has the same characteristics as the disordered monomeric form observed in aqueous solution.

## Introduction

α-Synuclein (αSyn) is a 140-residue protein whose aggregation process is strongly implicated in the pathogenesis of Parkinson’s disease and dementia with Lewy bodies [1], [2]. The monomeric form of this protein has been studied extensively in aqueous solution by a wide range of biophysical methods revealing a compact intrinsically disordered state without persistent secondary or tertiary structure [3]–[6]. Measurements of the hydrodynamic radius of this species have revealed that the structural ensemble is more compact than that expected for a random coil state [6], and NMR measurements of residual dipolar couplings and paramagnetic relaxation enhancements have identified weak interactions between the negatively charged C-terminal region (residues 100–140) and the positively charged N-terminal region (residues 1–100) and, in particular, with the hydrophobic NAC region (residues 60–90) [7]–[10]. It has also been observed, however, that in the presence of curved, anionic lipid surfaces the N-terminal region adopts essentially complete α-helical structure [11]–[13].

Although NMR spectroscopy is routinely applied to the study of structure and dynamics of proteins in vitro, recently the feasibility of performing high-resolution spectroscopic studies of proteins directly within living cells has been demonstrated – an approach termed ‘in-cell NMR’ [14]–[17]. One of the first systems to be observed was αSyn, and both the in-cell HSQC and the directly-detected CON spectra of αSyn expressed within bacterial cells have been reported to be similar to that of the isolated protein [18]–[21]. These observations indicated that αSyn remains intrinsically disordered within the cytosolic environment, and subsequent ^19^F NMR measurements of 3-fluorotyrosine chemical shifts also showed similar chemical shifts for intracellular αSyn when compared with the isolated protein [22]. The effect of N-terminal acetylation, a post-translational modification constitutively observed for αSyn in vivo, has also been investigated by in-cell NMR for αSyn co-expressed with the N-acetyltransferase NatB within *E. coli* cells [23]. While small chemical shift changes were observed in the isolated protein following N-terminal acetylation, consistent with the increase in the α-helical population in the first 12 N-terminal residues reported from in vitro studies [24] no additional changes were observed in the HSQC spectrum of the intracellular species [23].

In the present work, we have brought together advances in the in-cell measurements of chemical shifts with progress in the analysis of secondary structure populations in disordered proteins [25], to assess directly the conformation of αSyn within living cells. By determining a near-complete set of backbone chemical shift values of αSyn expressed within *E. coli* cells (limited by line broadening in the N-terminus), we find that αSyn populates a disordered conformation within the cell which, when compared with measurements of the isolated protein in dilute solution, is remarkably unperturbed by the highly crowded intracellular milieu.

## Materials and Methods

### Sample Preparation

Isolated ^13^C/^15^N-labelled αSyn was expressed and purified following established protocols [26]. NMR samples of *E. coli* BL21 (DE3) Gold cells (Stratagene) in which ^13^C/^15^N-labelled WT αSyn had been expressed (4 hr, 310 K) were prepared according to previously described protocols [27], [28] and resuspended as a ca. 30% (v/v) slurry in unlabelled M9 media (pH 7.4, 10% D_2_O, 0.001% DSS). In contrast to previous reports that αSyn may be expressed in the periplasm (depending on expression conditions) [18], [29], [30], in our hands we find that the expressed αSyn is localised entirely within the cytoplasm, and we have not detected any periplasmic fraction of αSyn that is released following osmotic shock using previously reported protocols [29]. The intracellular concentration of αSyn was determined by analysis of 1D ^1^H NMR spectra to be 1.7±0.3 mM. In comparison, the concentration of αSyn within dopaminergic neurons has been estimated to be several hundred micromolar [31].

### NMR Spectroscopy

NMR data were acquired at 277 K, on a Bruker Avance III spectrometer equipped with a TXI cryoprobe operating at 16.4 T (700 MHz), with a unidirectional gradient coil generating a maximum gradient of 0.55 T m^−1^. ^15^N-XSTE diffusion experiments [32] were measured in an interleaved manner between 3D experiments, and were analysed as previously described [28] in order to provide a continuous monitor of the sample integrity. BEST-HNCO experiments [33] were acquired with 8 scans per increment, a recycle delay of 200 ms, 40 points in the indirect ^15^N dimension with a spectral width of 26 ppm, and 80 points in the ^13^C dimension with a spectral width of 8 ppm. The acquisition time of each spectrum was 2.7 hours. BEST-HNCOCACB experiments [34] were acquired with 4 scans per increment, a recycle delay of 200 ms, 40 points in the indirect ^15^N dimension with a spectral width of 26 ppm, and 128 points in the ^13^C dimension with a spectral width of 70 ppm. The acquisition time of each spectrum was 2.5 hours. Spectra were referenced to DSS [35] then processed with linear prediction in both indirect dimensions and co-added using nmrPipe [36].

### Deconvolution of Inhomogeneous Broadening

Processed spectra in nmrPipe format were imported into MATLAB (R2011b, The MathWorks Inc.). 3D regions centered on a selected reference peak were identified and used to define a point spread function (PSF), which was used as input for Lucy-Richardson deconvolution [37]. HNCOCACB spectra were processed in stages, by generating subspectra containing only positive or negative peaks, which were deconvolved separately then recombined into a single deconvolved spectrum. The processed spectra were converted back into nmrPipe format and were analysed using CcpNmr Analysis [38].

## Results and Discussion

Monitoring the extent of protein leakage into the extracellular environment is a key challenge for in-cell NMR studies [39]. In the present work, the intracellular localization of the observed resonances was verified directly by observation of the restricted translational diffusion behavior characteristic of intracellular species [28]. One-dimensional heteronuclear (^15^N-XSTE) diffusion measurements [32] were recorded before and after all 3D NMR measurements, using a 300 ms diffusion delay as previously described [28]. Using this non-invasive method, when the fraction of extracellular αSyn exceeded 5%, data acquisition was halted and a fresh sample was prepared. NMR analysis of an expression time course (Figure S1) showed no discernable lag phase, indicating that the species being observed constitutes the major state of αSyn within the cell, in agreement with previous spectroscopic and biochemical analyses [21], [40].

To determine the HN, N, CO, Cα and Cβ backbone chemical shifts of intracellular αSyn a series of triple-resonance BEST-HNCO and BEST-HNCOCACB experiments were recorded [33], [34]. Each 3D spectrum was acquired in just 1–2 hours, which is an important factor as samples were typically found to contain significant (>5%) levels of extracellular species after just a few hours. Spectra were repeatedly acquired from a total of four samples, and were then summed to produce a final spectrum. Analysis of the chemical shift of the single histidine resonance (His50) showed that within 30 min of sample preparation, the intracellular pH was 6.2±0.1 (Figure S2), indicating acidification of the cytoplasm consistent with that observed previously under nutrient-depleted conditions (as pH homeostasis is an active process) [41]. Therefore, for comparison purposes HNCO and HNCOCACB spectra were also acquired for a sample of isolated (monomeric) αSyn in bulk solution at the same pH.

**Figure 1:**
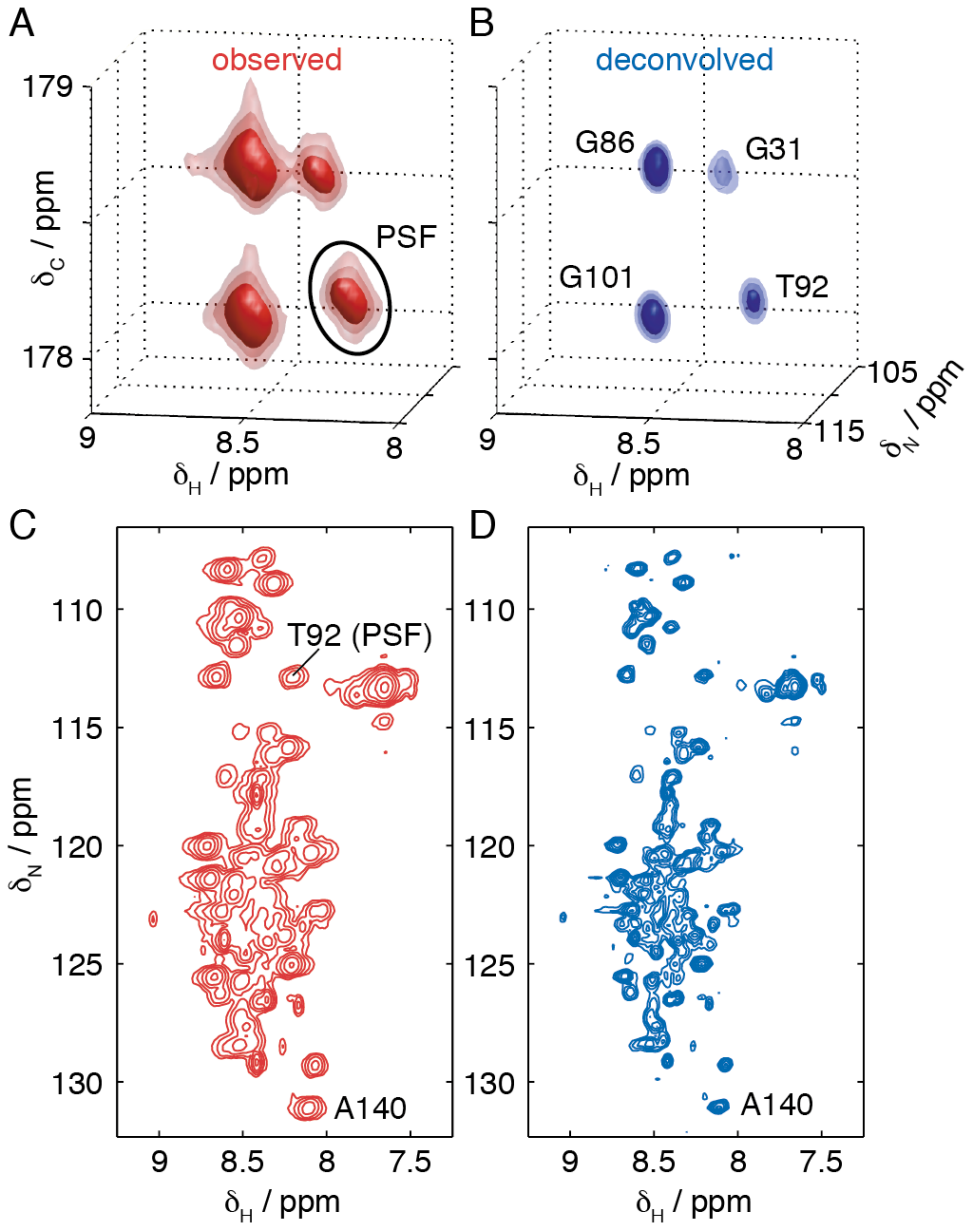
Multidimensional deconvolution of in-cell NMR spectra. HNCO spectra of αSyn (A) expressed within *E. coli* cells and (B) following deconvolution using the T92 reference peak indicated in (A). (C,D) ^1^H/^15^N projections of (C) the original HNCO spectrum and (D) the spectrum following deconvolution.

Because of the magnetically inhomogeneous environment characteristic of the dense cell samples studied here, having cell volume fractions of ca. 30%, αSyn resonances exhibit a strong inhomogeneous line broadening giving rise to diagonal lineshapes (Figure 1A). This effect can result in severe resonance overlap even within 3D spectra. As this broadening arises from variations in the magnetic field strength within the sample, its effect is constant on a ppm scale irrespective of the type of nucleus. This is therefore a particular problem for the determination of ^1^H chemical shifts in disordered states, due to the small chemical shift dispersion of ca. 1 ppm. The observed spectrum can be represented in the frequency domain as the ‘true’ spectrum convolved with a three-dimensional point spread function (PSF) that reflects the distribution of magnetic field strengths found across the sample, and which is therefore the same for all residues. We note that such line broadening cannot be eliminated using non-uniform sampling (NUS) methods, although for folded proteins where resonance overlap is a less significant problem NUS sampling schemes have been demonstrated to be very effective for the rapid acquisition of in-cell NMR spectra [16].

By analogy to one-dimensional reference deconvolution methods [42] and to image restoration in optical microscopy [43], we have estimated the PSF using the three-dimensional line shape of a well-resolved and isolated resonance (T92, Figure 1A) and used this in deconvolution algorithms to restore the original, unbroadened spectrum. A variety of deconvolution methods have been described, such as the Wiener filter [44] or maximum entropy methods [45]; in this instance we have found the iterative Richardson-Lucy algorithm [37] to be particularly effective. Regions and HN projections of the in-cell HNCO spectrum of a sample of αSyn expressed within cells before and after deconvolution are shown in Figure 1. The deconvolved spectra have a much more symmetric line shape, and show a significant reduction in linewidth (full width at half maximum), e.g. from ^1^H linewidths of 55±1 Hz to 31±1 Hz in the case of the A140 resonance (Figures 1C, 1D and S3). Such reductions in line broadening have greatly facilitated the spectral analysis that has been performed in this work.

**Figure 2:**
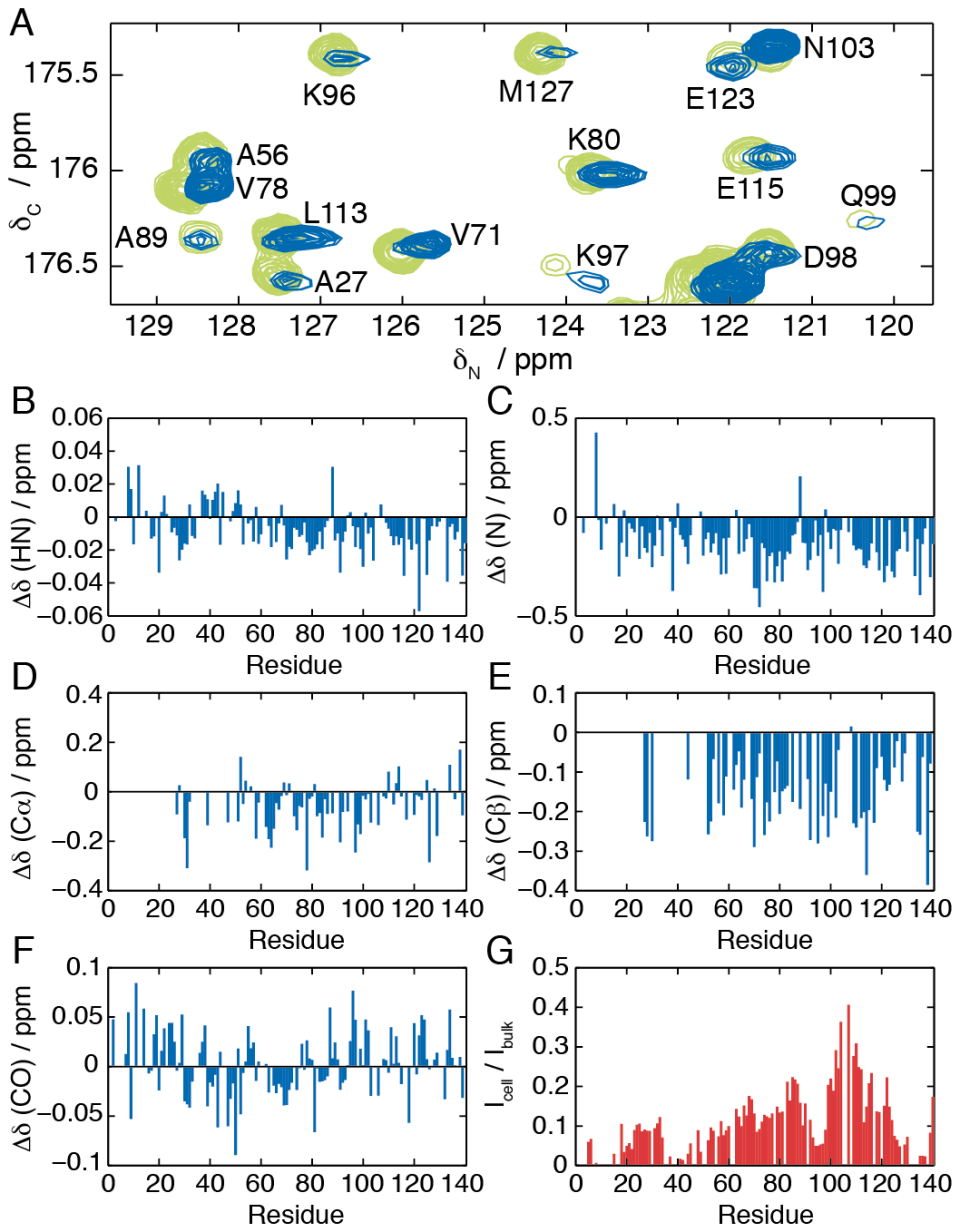
Analysis of backbone chemical shift changes and line broadening. (A) Overlay of ^13^C/^15^N slices through the HNCO spectrum of αSyn in bulk solution (green) and the deconvolved spectrum of αSyn expressed within cells (blue), showing resonances with ^1^H chemical shifts between 8.45 and 8.55 ppm. (B–F) Backbone chemical shift changes observed for intracellular αSyn relative to the protein in bulk solution. (G) Relative HNCO intensities of intracellular αSyn compared to αSyn in bulk aqueous solution.

The backbone resonances of intracellular αSyn were identified and attributed by comparison with spectra of the protein in bulk solution. Figure 2A shows a representative region of the deconvolved in-cell HNCO spectrum; while small shifts in peak positions are visible, the observed resonances generally overlay closely with those from the protein in bulk solution. The chemical shift differences determined in this manner are uniformly small (<0.05 ppm ^1^H, <0.4 ppm ^13^C, <0.5 ppm ^15^N) across the entire sequence (Figures 2B–F). We note that the plotted chemical shift scales are greatly magnified relative to the typical range of secondary chemical shift changes. As backbone chemical shifts are sensitive indicators of secondary structure [46], these results indicate that the average conformation of the protein does not change significantly within the cellular environment. However, while many nuclei exhibit both positive and negative changes in chemical shift, N and Cβ nuclei exhibit a small but systematic decrease in chemical shift values, which prompted a more detailed and quantitative analysis of the intracellular conformation and secondary structure formation.

**Figure 3:**
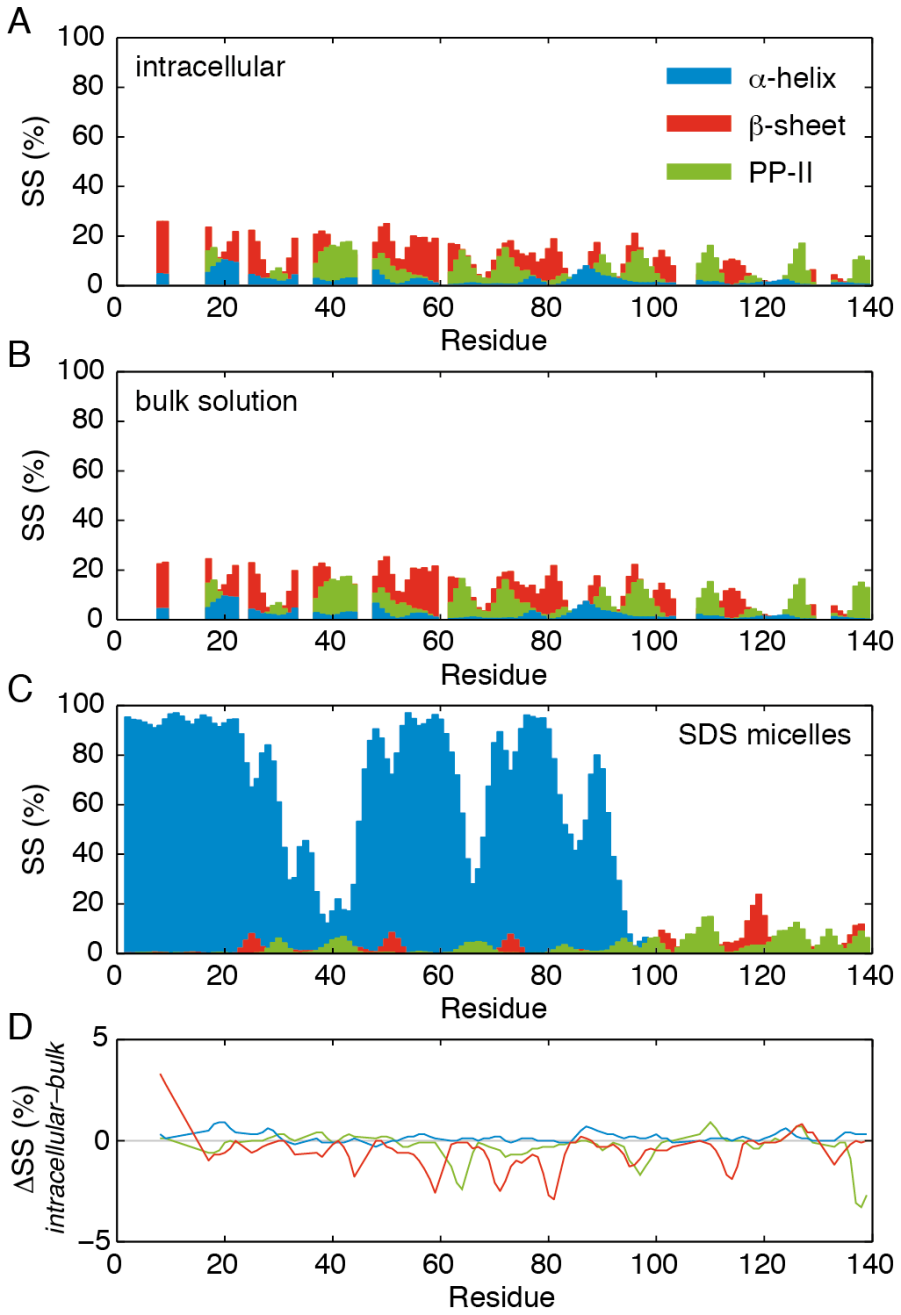
Comparison of secondary structure populations in isolated and intracellular αSyn. Secondary structure populations of αSyn calculated with the δ2D method [25] using the backbone chemical shifts of (A) intracellular αSyn, (B) monomeric αSyn in bulk solution at the same pH, and (C) SDS micelle-bound αSyn at pH 7.4. [12] (D) Differences in secondary structure populations between intracellular and bulk solution measurements.

To investigate these results in greater depth, the measured chemical shift values were translated into secondary structure populations by using the δ2D method [25] (Figure 3A). When backbone chemical shifts are fully determined, the δ2D algorithm can predict the population of secondary structures with an error of less than 10%. In some cases however, fewer chemical shifts per residue are available due to the rapid relaxation of the resonances, as discussed below. Nevertheless, quantitative comparisons between different states of a given system can still be achieved by using the same set of resonances for each residue, in such a way that systematic effects linked to the absence of chemical shifts are minimized. Secondary structure populations are shown for monomeric αSyn in bulk solution, measured at the same pH as found within the cell (Figure 3B), and for αSyn in an α-helical state formed in association with SDS micelles [12] (Figure 3C) – thought to be a mimic of α-helical states populated by αSyn in the presence of membranes. For every residue the difference in secondary structure content between intracellular αSyn and the protein in bulk solution is less than 5% (Figure 3D), and within the uncertainties of the δ2D method. No increases in α-helical content are observed that would be indicative of the rapidly reversible population of oligomeric [47], [48] or membrane-associated states [11]–[13], although our observations do not preclude the existence of an NMR-invisible membrane-bound sub-population of αSyn in slow exchange with the disordered state.

Although the chemical shift changes are small, and no change in the secondary structure content of αSyn is detectable within the cell, some differences are nevertheless apparent in the spectra, notably marked intensity changes in the HNCO spectrum of intracellular αSyn relative to the bulk solution state (Figure 2G). Decreased intensities are observed over much of the sequence, particularly in the region of the N and C-termini, and indeed as a result of this broadening no Cα and Cβ resonances could be detected between residues 1 and 26. Such peak broadening could arise from intermediate chemical exchange, indicating conformational fluctuations on a millisecond timescale, which has been observed previously in NMR studies of binding interactions involving αSyn [49], [50]. In particular, decreased intensities within the N-terminal domain (residues 1–100) suggests that interactions may be occurring with the cell membrane, as similar intensity changes have previously been observed for the isolated protein in the presence of model membrane systems [5], [13]. Within the cell however, line broadenings can also be due to transferred relaxation, as a result of weak and transient interactions with other large and slowly tumbling macromolecules within the crowded cellular environment; indeed the highly charged nature of the N and C-terminal regions of αSyn may result in a particular propensity for non-specific electrostatic interactions.

In summary, we have demonstrated that a multidimensional reference deconvolution strategy can substantially decrease the inhomogeneous line broadening associated with cellular samples, and the associated reduction in resonance overlap can greatly enhance the measurement of chemical shifts within crowded spectra. Using this approach, backbone chemical shifts have been measured for samples of αSyn expressed within bacterial cells, and used to evaluate secondary structure formation in this environment. Although selective reductions in peak intensity are observed, indicative of interactions with other components of the cell, only small chemical shift differences are observed compared with monomeric αSyn in bulk solution, indicating that in the crowded cytosolic environment the protein exhibits a disordered conformation whose secondary structure closely resembles that observed in studies of αSyn in dilute aqueous solution. More generally, given the increasingly recognized importance of intrinsically disordered proteins or domains in many cellular processes, we believe that the approach we have described here will become an important method to investigate the structure and behavior of such molecules directly within the cellular environment.

## Supporting Information

Figure S1 Timecourse of αSyn expression in *E. coli* BL21 (DE3) Gold cells, measured as the integrated amide intensity in the first increment of a diffusion-edited ^15^N XSTE-HSQC experiment, where the gradient strength *G* = 0.52 T m^−1^, the gradient pulse length δ = 4 ms, and the diffusion delay Δ = 300 ms. Cell samples were diluted to a constant density (OD_600_ = 40) prior to measurement.
(PDF)

Figure S2 pH dependence of His50 ^1^H chemical shift, measured for αSyn in bulk solution at 277 K (blue) and fitted to a modified Henderson-Hasselbalch equation in order to estimate the cytosolic pH of cell samples in which αSyn had been expressed (red).
(PDF)

Figure S3 **Cross-sections through A140 HNCO resonances before and after PSF deconvolution, for the determination of ^1^H linewidths.**
(PDF)
