# Supplementary figures and images for: In-Cell NMR Characterization of the Secondary Structure Populations of a Disordered Conformation of α-Synuclein within E. coli Cells
Source: PLoS One. 2013 Aug 26;8(8):e72286. doi: 10.1371/journal.pone.0072286 (PMC3753296; doi:10.1371/journal.pone.0072286)

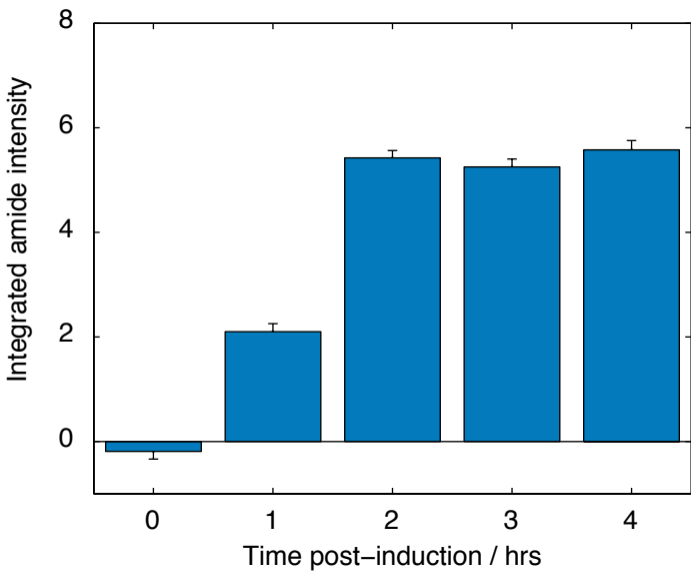

Supplement: Figure S1 — Timecourse of αSyn expression in E. coli BL21 (DE3) Gold cells, measured as the integrated amide intensity in the first increment of a diffusion-edited 15N XSTE-HSQC experiment, where the gradient strength G = 0.52 T m−1, the gradient pulse length δ = 4 ms, and the diffusion delay Δ = 300 ms. Cell samples were diluted to a constant density (OD600 = 40) prior to measurement. (PDF) [file pone.0072286.s001.pdf]

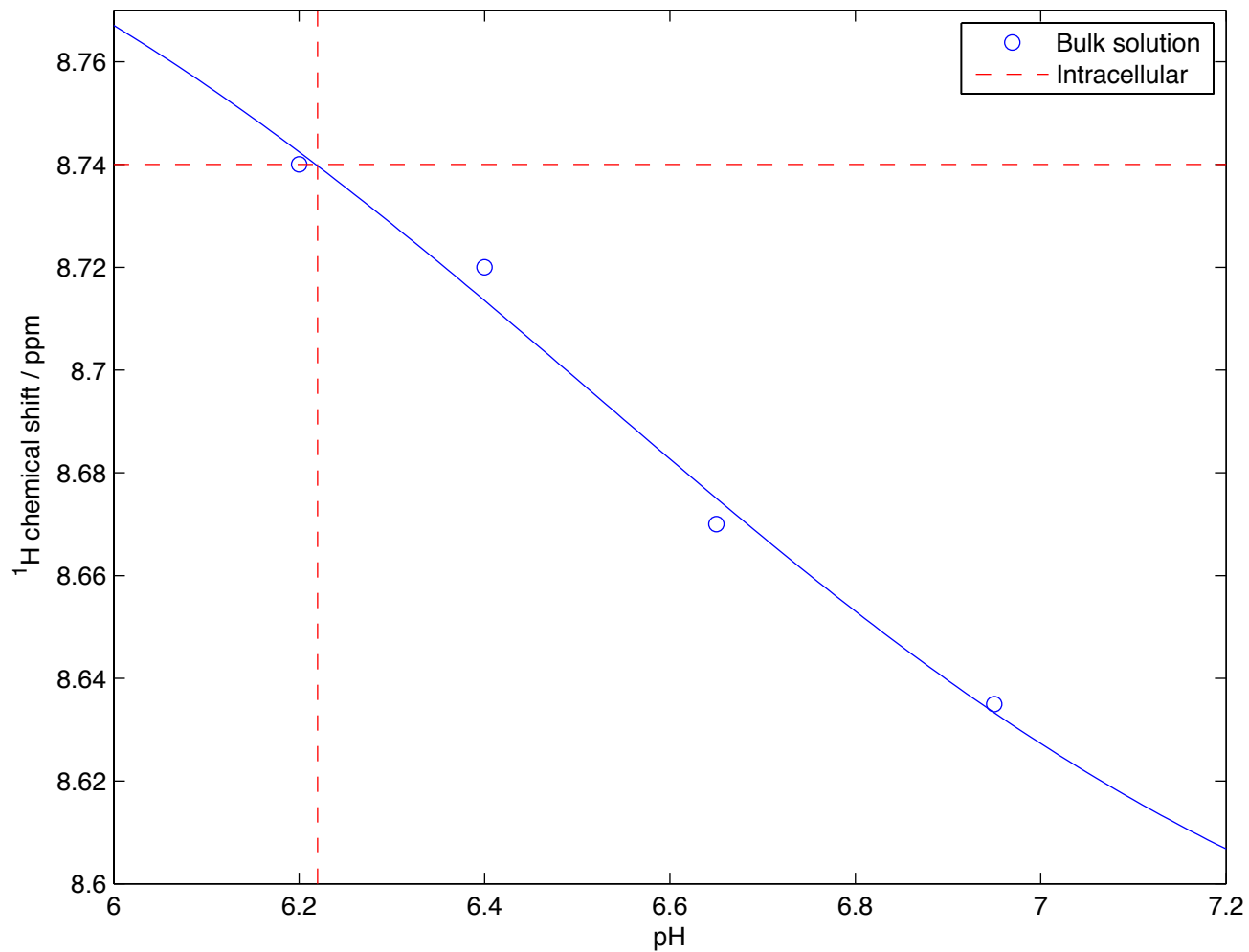

Supplement: Figure S2 — pH dependence of His50 1H chemical shift, measured for αSyn in bulk solution at 277 K (blue) and fitted to a modified Henderson-Hasselbalch equation in order to estimate the cytosolic pH of cell samples in which αSyn had been expressed (red). (PDF) [file pone.0072286.s002.pdf]

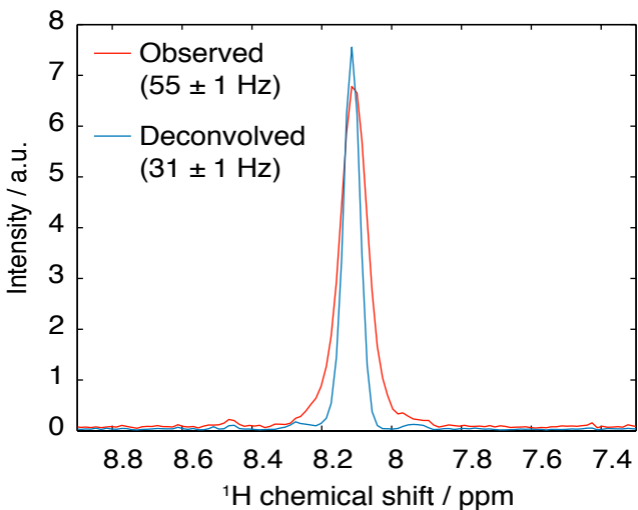

Supplement: Figure S3 — Cross-sections through A140 HNCO resonances before and after PSF deconvolution, for the determination of 1H linewidths. (PDF) [file pone.0072286.s003.pdf]
